# The senescent vision: dysfunction or neuronal loss?

**DOI:** 10.18632/aging.101734

**Published:** 2018-12-26

**Authors:** Francisco M. Nadal-Nicolás, Manuel Vidal-Sanz, Marta Agudo-Barriuso

**Affiliations:** 1 Retinal Neurophysiology Section, John Edward Porter Neuroscience Research Center, National Eye Institute, National Institutes of Health, Bethesda, MD 20892, USA; 2 Dpto de Otalmología, Facultad de Medicina, Universidad de Murcia e Instituto Murciano de Investigación Biosanitaria Virgen de la Arrixaca, Murcia, Spain

**Keywords:** aging, retina, retinal ganglion cells, melanopsin, photoreceptors, microglia, astrocytes

Aging is the most common degenerative process consequence of multiple cumulative mechanisms. The permanent effects of senescence are complex and heterogeneous; genetic heritage, nutrition, behavior, physic exercise and environment factors are tangled during the course of life. Aging is associated with chronic degenerations [1] and so, it is difficult to identify and characterize the intrinsic components of aging *per se*.

Visual aging is linked to a decline in functional activity causing lower visual acuity, lower contrast sensitivity and impaired dark adaptation. However, although it has been reported that the age-related visual impairment is mainly due to a neuronal malfunction together with cell loss, the subjacent and specific reasons of aging are still uncertain. How, and at what level, are the diverse neuronal populations affected? And how much are other retinal players involved?

**Figure 1:**
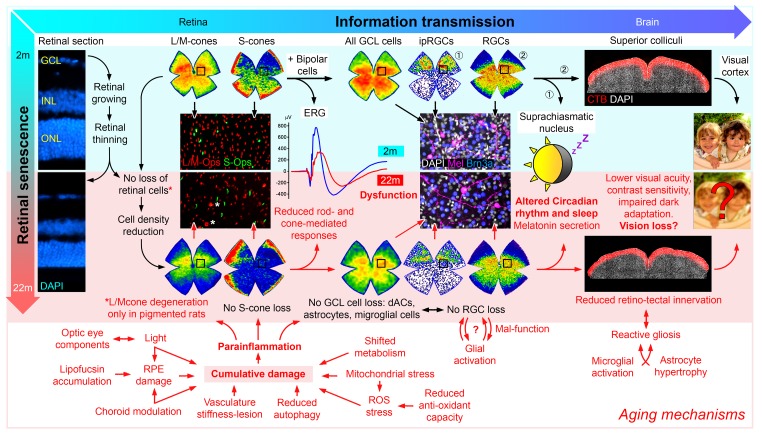
**Aging effects on the visual pathway.** In the retina, the continuous growth decreases both the retinal thickness and the density of the different retinal populations (details in [2]), however, although no neuronal loss was observed in aged retinas (except, L/M-cones in pigmented rats), the vision forming and the non-vision forming information, that reaches the brain, are likely affected by aging [7].

By characterizing retinal aging in experimental animals (pigmented and albino rats) under controlled and healthy conditions, we found that the retinal function, as measured with full field electroretinograms, decreased ~50% at 22-months compared with 2-month-old rats [2]. Whether neuronal malfunction or cell loss is mainly responsible for this reduced functionality is still an open question, even though structural changes in the optical components may contribute to this reduction. Interestingly, several studies suggest cell loss based on the retinal thinning that occurs with aging [3]. However, although when we measured the retinal layers *in vivo* we observed a decrease in thickness ~14% [2], we also saw that the constant retinal growth was responsible for the retinal thinning, since volumetric and quantification analyses indicated that the thinning did not involve neuronal loss (except for L/M-cones in pigmented rats) [2], (Figure 1).

The retina is a highly organized and specialized tissue. The light-sensitive photoreceptors are essential for an effective signal transduction and to initiate the efficient transmission of impulses through the retina. They are vulnerable to light-induced damage and many publications have shown the degeneration of outer segments during aging. Interestingly, we observed loss of L/M-cones in the central retina of 22-months-old pigmented rats [2], comparable to the photoreceptor-loss in the macular region of aged human retinas [4]. The central retina probably receives greater light exposure triggering different metabolic requirements that increases metabolic stress. In fact, a deficiency in DNA repair enzymes, damage induced by excitatory amino acids, specific age-related metabolic changes [5], a general decline in autophagy activity, and reduced energy production by mitochondrial metabolism collectively result in oxidative stress that may affect photoreceptor functionality. All that in addition to lipofuscin accumulation, morphological alterations and damage in the retinal pigmented epithelium [4] accompanied by a para-inflammatory response [6] are the signature signs of aging in the retina.

The reduced b-wave and increased implicit time observed in aged animals may be also explained by reduced function of bipolar cells [2]. But these results are difficult to interpret because, although we cannot rule out synaptic alterations, the b-wave would also reflect the corresponding diminished photoreceptor activity. Later, the signal reaches the retinal ganglion cells (RGCs), whose degeneration is widely accepted in aged retinas [3]. Our results show a decrease in RGC density, but not in the number of orthotopic or displaced RGC [2]. As above-mentioned, the decreased density is due to retinal growth, and it has been shown that animals with a reduced food intake maintain their RGC density, because the area could remain constant. Obviously, the fact that the number of RGCs does not change with age does not necessarily mean that these cells are healthy or functional. Furthermore, aged microglia show increased dystrophic signs in their morphology in the retina, i.e. reduced dendritic arbors with slow migration capability [7]. We cannot elucidate whether microglial cells become pathogenic with age, or whether they are trying to keep the changing retinal homeostasis under control.

Although our results did not show loss of ip-RGCs in aging retinas [2], morphological changes like reduced dendrites or even lower melanopsin expression have been recently reported, and this could explain an impaired function since circadian rhythm and sleep are strikingly influenced by aging [8].

Lastly, before the images are formed in the visual cortex, the information reaches the superior colliculi (SCi), the main retinorecipient area in rodents. In aged animals, there was a slight reduction of RGC projections to the SCi, as well as astrocyte hypertrophy and microglial cells activation, both symptoms of stress or degeneration [2]. This may be a compensatory mechanism to maintain a lower neuronal activity of the aged neurons or to maintain the disrupted tissue homeostasis [7].

To preserve visual function, the eyes and brain require precisely tuned machinery. Any of the above-mentioned changes related to aging, including synapse remodelling or neuronal loss in response to age may contribute or play a crucial role in the continuous and irreversible decline in vision. Importantly, age may end causing a partial or complete distorted image formation, more so in a timeframe where our lifespan is increasing. So, could this retinal dysfunction be prevented or restored?
